# The relationship between visual ability assessment and competitive boxing performance in female amateur boxers

**DOI:** 10.3389/fphys.2025.1639227

**Published:** 2025-08-06

**Authors:** Youlin Xiao, Huihui Zhong, Dongxu Gao, Mingnan Zhuang, Yuankun Long, Qiang Wei, Dexin Wang, Peng Zhang, Zihao Zhao, Chao Chen

**Affiliations:** ^1^ Sports College, Dalian University, Dalian, China; ^2^ Department of Physical Education, Suzhou Vocational University, Suzhou, China; ^3^ Division of Sports Science and Physical Education, Tsinghua University, Beijing, China; ^4^ College of Sports Training, Tianjin University of Sport, Tianjin, China; ^5^ College of Sports Training, Shanghai University of Sport, Shanghai, China; ^6^ Sports College, Jiamusi University, Jiamusi, China; ^7^ Higher Vocational College, Shaanxi University of International Trade, Xi’an, China

**Keywords:** punching performance, eye-hand coordination, reaction time, female boxers, sports vision

## Abstract

**Introduction:**

The relationship between visual abilities and punching performance has received considerable attention in sports science, but research on female amateur boxers remains limited. This study investigates the correlation between visual‑motor abilities and punching performance in female amateur boxers.

**Methods:**

A total of 26 trained female boxers participated in the study, and their visual abilities were assessed using the Senaptec Sensory Station. The visual ability tests included measures of visual clarity (VC), contrast sensitivity (CS), depth perception (DP), perceptual span (PS), multiple object tracking (MOT), reaction time (RT), eye‑hand coordination (EHC), and Go/No‑Go (GNG). Punching performance was analyzed by evaluating the hit percentage (%Hit) in the National Boxing Championship.

**Results:**

Pearson correlation analysis showed that punching accuracy (%Hit) was strongly correlated with EHC, RT, PS, and DP. Stepwise regression analysis confirmed that these visual abilities significantly predicted punching performance, with EHC, RT, PS, and DP explaining 93.1% of the variance in %Hit.

**Discussion:**

The results highlight the significant role of visual motor abilities in enhancing the punching performance of female boxers. The study suggests that training programs targeting these visual abilities, especially EHC, RT, and DP, could improve boxing performance. This research provides valuable insights into the role of vision in female boxing and suggests future directions for visual training in combat sports.

## 1 Introduction

Sports vision is an emerging field aimed at establishing the relationship between visual abilities and athletic performance (Laby et al., 2011). It involves not only visual acuity but also multiple aspects such as visual information processing, reaction speed, and hand-eye coordination (Balaji et al., 2021). Specifically, visual abilities include visual sharpness, depth perception, contrast sensitivity, and visuomotor reaction speed (Ramyarangsi et al., 2024). These abilities enable athletes to effectively observe and respond to visual stimuli during sports activities. Approximately 80% of environmental information in sports is obtained through an athlete’s visual system and its components (Ripoll et al., 1995). Furthermore, vision and visual processing are widely recognized as crucial components of successful competitive performance (Erickson et al., 2011). In various sports, the close integration of visual perception and motor action is essential for athletic performance, directly influencing an athlete’s ability to execute precise and complex movements, and is considered a key limiting factor for achieving elite performance levels (Klostermann et al., 2020; Saito et al., 2020).

Visual-motor abilities play a critical role across different sports disciplines, with the specific visual skills required varying according to the characteristics of each sport (Ramaja and Hansraj, 2023). For example, baseball is a sport with extremely high visual demands, requiring athletes to accurately judge the speed, spin, and trajectory of a pitched ball within a very short timeframe, necessitating excellent dynamic visual acuity, depth perception, and visual reaction speed (Gohdo et al., 1997). Soccer players require superior dynamic vision, peripheral awareness, and hand-eye coordination to observe teammates and opponents while moving at high speed, enabling precise passing and shooting (Millard et al., 2020). Similarly, ice hockey demands rapid visual reactions and accurate judgments to make correct decisions during fast-paced play (Poltavski and Biberdorf, 2015). Sports vision typically encompasses visual clarity, contrast sensitivity, depth perception, visual field, target tracking, target acquisition, near-far rapid focusing, hand-eye coordination, Go/No-Go responses, and reaction time (Erickson, 2021; Regan and Gray, 2001). These studies support the clear link between enhanced visual abilities and improved athletic performance (Wu et al., 2024). Moreover, athletes generally exhibit superior visual abilities compared to non-athletes, and elite athletes typically outperform their less skilled counterparts in these visual functions (Gao et al., 2015).

In boxing, athletes must execute offensive and defensive actions, as well as transitions between them, almost instantaneously. Experienced boxers typically throw a punch within approximately 402–405 milliseconds (Stanley et al., 2018). This demands sustained attention and keen visual observation of the opponent, highlighting the critical role of visual abilities in boxing. Research has shown that superior visual skills contribute to faster reaction times and greater accuracy in boxers (Baghel et al., 2024). Visual abilities integrate perception, attention, anticipation, inhibitory control, and cognitive flexibility. Effective visual search strategies and heightened sensitivity to key visual cues enable boxers to better focus their attention, leading to quicker and more precise responses (Zahra et al., 2015). Thus, visual abilities play a significant role in boxing performance, and sports vision appears to be a key factor influencing boxer performance (Wu et al., 2024).

There are numerous physiological differences between male and female athletes that impact athletic performance, muscle characteristics, and training adaptations (Chino et al., 2018). From a neuroanatomical perspective, studies indicate that males generally have larger brain volumes, whereas females tend to have more developed frontal lobes and limbic systems (Li, 2014). This may partially explain why males often excel in certain spatial tasks, while females perform better in other tasks, such as language and memory (Li, 2014). Regarding visual function, a study comparing male and female judo athletes’ neurodynamics found that male athletes demonstrate superior efficiency in visual information processing (Jin et al., 2022). Different sports impose distinct physical demands, resulting in varied advantages for male and female athletes across disciplines. In boxing, female fighters generally have lower muscle mass compared to male fighters in the same weight class (Chaabène et al., 2015). However, females tend to exhibit advantages in endurance metabolism, such as greater fatty acid oxidation efficiency, which may influence strategic decisions in multi-round bouts (Savikj and Zierath, 2020).

In terms of visual abilities, one study found that female boxers exhibit superior visuomotor coordination. In eye-hand coordination tests, females achieved a 15.4% higher accuracy rate in performing complex combination punches compared to males (Çetin et al., 2018). Conversely, males demonstrated an advantage in responding to explosive visual stimuli; the activation latency of the motor cortex in males when responding to sudden visual threats was 140 ms shorter than that of females, a rapid stress response that can enhance defensive success in close combat (Jansen et al., 2021). Another study using eye-tracking data across three consecutive rounds of matches revealed that the saccadic frequency of male boxers decreased by 23% toward the end of the second round, whereas females showed only a 9% decrease (Shtanagey et al., 2021). This may be related to a higher proportion of slow-twitch fibers in the extraocular muscles of females, allowing more stable ocular motor control during sustained visual search. Most analyses of gender differences in boxing focus on body composition and muscular aspects, with few studies providing a comprehensive and systematic comparison from the perspective of visual function. One major reason for this gap is the limited amount of foundational research on the visual abilities of female boxers.

Over time, the status of women in the Olympics has steadily increased (Miragaya, 2006), yet research has not advanced at the same pace as the rise of women’s boxing. Dinçe et al. emphasized that boxers need to rapidly process visual information and respond accordingly, with visual reaction time being crucial in combat sports such as boxing and wrestling (Dinçer et al., 2022). Laby et al. compared the visual abilities of Olympic athletes and noted that boxers require a comprehensive set of visual skills, among which reaction time and hand-eye coordination are paramount (Laby et al., 2011). These studies represent some of the few investigations related to visual abilities in boxing; however, they predominantly focus on male boxers. Research on the relationship between visual abilities, athletic performance, and competitive outcomes in female boxers remains largely absent. Therefore, more research focused on female athletes is needed to gain a fuller understanding of the role and impact of vision in boxing.

Since the inception of the modern Olympic Games, changes in women’s boxing competition rules have had a significant impact on athletes’ competitive strategies. In 2013, the International Boxing Association (AIBA) implemented major revisions to the rules of women’s amateur boxing, adopting the “10-point must” scoring system. This system requires judges to evaluate bouts based on specific criteria to determine the winner (Thomson et al., 2013). These criteria include valid target areas struck, the number of effective punches landed, the ability to control the match through technique and tactics, and overall competitiveness. Among these, the number of valid target hits and effective punches are the most critical factors in scoring decisions (Davis et al., 2013), meaning that not only the act of hitting but also the effectiveness and accuracy of the punches are taken into account. Dunn, Lenetsky, and colleagues identified punch effectiveness (hits%) as a key indicator of scoring success (Dunn et al., 2017; Lenetsky et al., 2013), which is also regarded as a major hallmark of boxing dominance (Dunn et al., 2017). Therefore, female boxers should prioritize achieving higher punching accuracy in training, with vision playing a crucial role in providing the precise and rapid information necessary for accurate responses (Martínez De Quel and Bennett, 2019).

This study aims to reveal the crucial role of visual-motor skills in the performance of female boxers. It advances theoretical research on gender differences in visual-motor abilities and offers personalized training guidance tailored to female boxing athletes, addressing the often-overlooked visual training needs in their current programs. The findings provide a scientific basis for enhancing the competitive level of female boxers, promote the development of women in the sport, and contribute to further research on visual-motor skills and gender differences. We hypothesize that certain variables of sports visual ability are positively correlated with punching performance in female amateur boxers.

## 2 Methods

### 2.1 Participants

A total of 26 trained female amateur boxers participated in this study (age: 24.69 ± 5.48; height: 170.81 ± 6.66; weight: 67.07 ± 7.39; experience: 7.19 ± 2.51). All boxers met the requirements of amateur boxing competition rules, including weight categories ranging from flyweight to super middleweight [50 kg (n = 3),54 kg (n = 1),57 kg (n = 4),60 kg (n = 5),66 kg (n = 3),75 kg (n = 9)]. Inclusion criteria were as follows: (a) at least 5 years of professional boxing training; (b) participation in at least 3 national competitions; (c) placement within the top three in at least one national competition; (d) normal visual acuity and function without ophthalmic diseases or corrective treatments; (e) no prior visual skills training. All participants were fully informed about the study’s purpose, benefits, and potential risks. Written informed consent was obtained from all participants.

### 2.2 Materials

#### 2.2.1 Sports visual ability assessment

This study employed the Senaptec Sensory Station (Senaptec Inc., Beaverton, Oregon, USA) to evaluate 10 visual abilities. This device has demonstrated strong reliability [for detailed information on the reliability and validity of the protocol, see Erickson et al. (2011), Wang et al. (2015), Poltavski and Biberdorf (2015), Knöllner et al. (2022), and Appelbaum and Erickson (2016)]. The Senaptec Sensory Station consists of a series of ten computerized visuomotor tasks, each designed to assess a specific aspect of participants’ visual-motor abilities. The measures for each visual ability are summarized in Table 1.

**TABLE 1:** Detailed description of motor visual ability test.

Test indicators	Detailed methods	Evaluation criteria
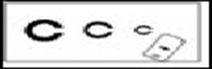 Visual Clarity VC Task	The participant holds a mobile device and stands 3 m away from a tablet screen. They judge the direction of the gap in a C-shaped figure displayed on the tablet and swipe the corresponding direction on the mobile device. Monocular vision is tested first for the left and right eyes, followed by binocular vision	The smaller the figure size at which the participant can accurately judge the direction, the better. The test metric is expressed in logMAR units, where lower values indicate better performance. A 5-point scoring system is used, with 5 being the highest score
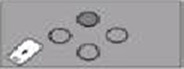 Contrast Sensitivity CS Task	The participant holds a mobile device and stands 3 m from the tablet. Four black circles appear on the tablet screen; one circle, randomly oriented, contains concentric circles of varying shades. The participant must identify this circle and swipe in the corresponding direction on the mobile device	As accuracy improves, the contrast within the concentric circles becomes less distinct. The test metric is measured in logCS (log Contrast Sensitivity), with higher values indicating better sensitivity
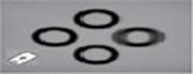 Depth perception DP task	Standing 3 m from the tablet, the participant sees four black circles on the screen; one randomly displays a stereoscopic effect. The participant must find this circle and swipe in the corresponding direction on the mobile device. Binocular vision is tested first, followed by separate tests for the right and left eyes	As accuracy increases, the contrast and stereoscopic effect of the target circle become less pronounced. The test metric is in arcseconds (arcsec), where smaller values indicate better stereoscopic vision
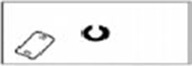 Near-far Fast N/FQ Mission	The participant stands 3 m from the tablet, holding the mobile device so that its top is 40 cm below the bottom of the tablet screen. During the test, C-shaped figures alternate between the tablet (far) and mobile device (near). The athlete switches focus between near and far every 30 s to judge the gap direction, then quickly swipes in the corresponding direction on the mobile device	Faster judgment speed and higher directional accuracy are preferable. Test metrics include the number of swipes within 30 s (higher is better) and reaction times for near and far focus shifts measured in milliseconds (ms), where lower values indicate faster responses
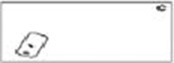 Target acquisition TC mission	The participant holds a mobile device and stands 3 m from the screen, aligning the blue reference line in the center of the screen with their line of sight, focusing on the center point. C-shaped figures randomly appear in the four corners of the screen; the participant judges the gap direction and swipes the corresponding direction on the mobile device	Reaction times measured in milliseconds (ms); faster speeds correspond to better performance
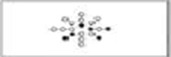 Perception span PS task	The participant stands 60 cm from the tablet screen, with eyes level to the center of the screen. Radial circles emanate from the center, some of which briefly flash black dots at their centers. The athlete must identify and tap the circle containing the black dot	The number of circles and black dots increases continuously across a wider range. Scores are based on the cumulative number of correct identifications, with higher scores indicating better performance
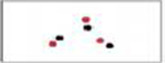 Multi-target tracking MOT mission	The participant stands 60 cm from the tablet screen, eyes level with the screen center. Several groups of spheres appear, each containing two black spheres. One sphere briefly changes to red then quickly returns to black before rotating randomly clockwise or counterclockwise. After rotation stops, the participant must identify the sphere that initially turned red in each group	Test metrics include the number of correctly tracked targets, tracking speed (degrees per second, °/s), percentage-based scores, and composite scores. Higher values indicate better performance
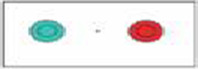 Response Time RT Task	The participant stands 60 cm from the tablet screen, eyes level with the screen center. Radial circles emanate from the center; some flash black dots briefly. The participant must identify and tap the circles containing the black dots	Similar to above, with increasing numbers and range of circles and black dots, scoring is based on cumulative correct judgments; higher scores indicate better performance
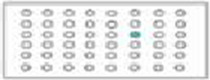 Hand-eye coordination EHC task	The athlete stands 60 cm from a large screen, raising the screen’s midline to align with or slightly above the arms to avoid obstructing peripheral vision. The screen displays 8 columns by 10 rows of hollow rings. One ring randomly changes to blue-green, and after the participant clicks it, another ring appears at a random position. The goal is to click as many as possible quickly and accurately within the allotted time	Metrics include total time, average reaction time, central region reaction time, and peripheral region reaction time all measured in milliseconds (ms), with lower values indicating better performance
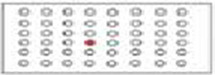 Go/No Go (GNG) task	The athlete stands 60 cm from the large screen, with the screen midline aligned at or slightly above arm level to prevent interference with peripheral target tapping speed. The screen shows 8 columns of circles identical to those in the eye-hand coordination test. Green and red dots appear randomly on the circles; green dots require a quick tap, while red dots must not be tapped	Metrics include overall score, number of correct taps (higher is better), and number of incorrect taps (lower is better)

#### 2.2.2 Boxing performance analysis

Each boxer’s matches at the National Boxing Championship were recorded using a video camera (Sony FDR-AX700, China). All participants competed in this tournament. The matches were analyzed using high-definition slow-motion playback, adjusted in 0.1-s increments to ensure accurate observation. All match analyses were conducted by the same certified boxing performance analyst. To assess intra-rater reliability, five matches were randomly selected for re-analysis. Additionally, a second experienced analyst independently analyzed these same five matches to determine inter-rater reliability. The number of punches thrown, the number of successful hits, and the number of missed punches were meticulously recorded for each round and match. The key performance metric selected for this study was punch accuracy, calculated as the percentage of punches landed out of total punches thrown (% Hit). For further details on performance parameters and boxing performance analysis methods, refer to Thomson et al. (2013), Davis et al. (2013), Davis et al. (2015), Davis et al. (2016), Davis et al. (2018), and Dunn et al. (2017).

### 2.3 Procedure

This study involved two assessments. First, the 10 sports visual ability tests were administered 1 week before the competition; all tests were completed on the same day. The second assessment involved analyzing the boxers’ in-ring punching performance during the competition. The sports visual ability tests were conducted in the Sports Visual Performance Laboratory. All boxers completed the visual ability assessment within 1 week prior to the National Boxing Championship. Upon arrival at the laboratory, informed consent was obtained from each participant. Subsequently, descriptive information was collected, including name, age, sex, height, and handedness/footedness. Additional information such as sport type and background (level, primary/secondary sport, position), vision correction status, and concussion history was also recorded. The visual ability assessment took approximately 25 min to complete. Each of the 10 computerized visuomotor tasks began with an animated demonstration, followed by three practice trials and then the formal test. Participants were instructed to guess if uncertain during the recognition tasks. Data collection for boxing performance analysis took place during the 2024/2025 season. Matches from the National Boxing Championship during this season were recorded using video cameras. All matches were analyzed by a certified boxing performance analyst, who holds boxing referee qualifications and extensive experience in match performance analysis. Prior to the analysis, meetings were held with elite boxing coaches to identify the key variables to be recorded during analysis, including punches thrown, hits, and misses.

### 2.4 Statistical analysis

Data were analyzed using IBM SPSS Statistics (version 27.0) and are presented as mean ± standard deviation (SD). The Shapiro-Wilk test was used to assess the normality of the data. Pearson correlation coefficients (r) and their corresponding 95% confidence intervals (95% CI) were calculated to evaluate the linear relationships between visual abilities and punching performance. Multiple regression analysis was conducted to examine the associations between visual ability variables and punch accuracy in boxers. The assumptions of normality, linearity, homoscedasticity, and independence for the regression model were verified through distribution and residual analyses. Intra-class correlation coefficients (ICC) were used to assess the consistency of repeated analyses of five randomly selected matches, as well as inter-rater reliability between analysts. Interpretation of correlation coefficients followed Cohen’s guidelines: r = 0 indicates no correlation; 0 to 0.3, weak; 0.3 to 0.5, moderate; 0.5 to 0.7, strong; and 0.7 to 1, very strong. The adjusted coefficient of determination (*R* ^2^) was used to evaluate model fit. The significance level was set at p < 0.05. Post hoc power analysis was conducted using G*Power 3.1 based on the observed effect size (Cohen’s f^2^) and α = 0.05 to determine the sensitivity of the model to Type II error (Faul et al., 2009).

## 3 Results

**FIGURE 1:**
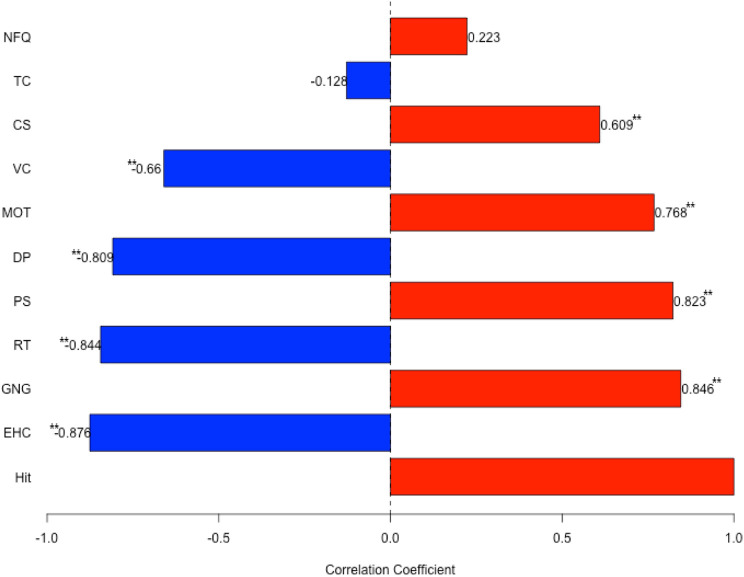
Correlation coefficients between boxers’ visual ability and punching performance. a b *p < 0.05. **p < 0.01.

All boxers successfully completed the testing tasks and competition. The average intraclass correlation coefficient (ICC) used to assess the consistency between the two analyses was 0.994, indicating excellent agreement. Additionally, the inter-rater ICC assessing the consistency between primary analysts was 0.915, which falls within an acceptable range. Table 2 presents the descriptions of the boxing performance analysis variables. Table 3 lists the descriptive statistics. Figure 1 illustrates the Pearson’s correlation coefficients (r) and their 95% confidence intervals (95% CI) between female boxing performance and visual ability parameters. Table 4 shows the results of the multiple stepwise regression analysis.

**TABLE 2:** Description of boxing performance analysis variables.

Variable	Unit	Description
Punches Thrown	Number	Total number of punches thrown
Hit	Number	A punch that hits the target area
Miss	Number	A punch that misses the target area
%Hit	%	Number of hits to the target area as a percentage of total punches thrown

**TABLE 3:** Descriptive analysis of anthropometrics, visual ability tests, and punch accuracy.

Parameter	M ± SD N = 26
Personal data	
Age(y)	24.69 ± 5.48
Height (cm)	170.81 ± 6.66
Weight (kg)	67.07 ± 7.39
Experience(y)	7.19 ± 2.51
Visual ability variables	
EHC(s-)	50.57 ± 4.15
GNG (scoer+)	6.32 ± 1.40
RT (ms-)	302.82 ± 24.28
PS(score+)	41.21 ± 8.71
DP (arcsec-)	167.90 ± 56.85
MOT (score+)	0.70 ± 0.10
VC(logMAR-)	−0.03 ± 0.08
CS(logCS+)	1.67 ± 0.26
TC (ms-)	271.32 ± 83.19
NFQ (score+)	16.15 ± 3.75
Punch variables	
Punch accuracy (%Hit)	25.25 ± 7.76

VC, visual clarity; CS, contrast sensitivity; DP, depth perception; NFQ, near far quickness; TC, target capture; PS, perception span; MOT, multiple object tracking; EHC, Eye-Hand Coordination; GNG, Go/No Go; RT, reaction time; + = higher is better; − = lower is better.

**TABLE 4:** Standardized regression to explain the punch performance (%Hit).^a^

Variable	ß	CI for ß	P
EHC	−0.304	−0.931 to −0.206	0.004**
RT	−0.309	−0.152 to −0.045	0.001**
PS	0.231	0.046 to 0.366	0.014*
DP	−0.278	−0.06 to −0.016	0.002**
Modelt fit	Adjusted *R* ^2^ = 0.931		

^a^ %Hit = hit rate of punches thrown; EHC, Eye-Hand Coordination; RT, reaction time; PS, perception span; DP, depth perception; βa = estimated standardized regression coefficient.

*p < 0.05.

**p < 0.01.

Correlation analysis revealed that among all visual ability variables, boxing performance (%Hit) was moderately correlated with visual clarity (VC; *r* = −0.66, *p* < 0.001) and contrast sensitivity (CS; *r* = 0.609, *p* = 0.004). Strong correlations were found between %Hit and depth perception (DP; *r* = −0.809, *p* < 0.001), as well as peripheral speed (PS; *r* = 0.823, *p* = 0.009). A strong correlation was also observed between %Hit and multiple object tracking (MOT; *r* = 0.768, *p* = 0.049). Very strong correlations were identified between %Hit and eye-hand coordination (EHC; *r* = −0.876, *p* < 0.001), go/no-go (GNG; *r* = −0.846, *p* < 0.001), and reaction time (RT; *r* = −0.844, *p* < 0.001). No significant correlations were found between %Hit and near-far quickness (NFQ) or target capture (TC) (Figure 1).

**FIGURE 2:**
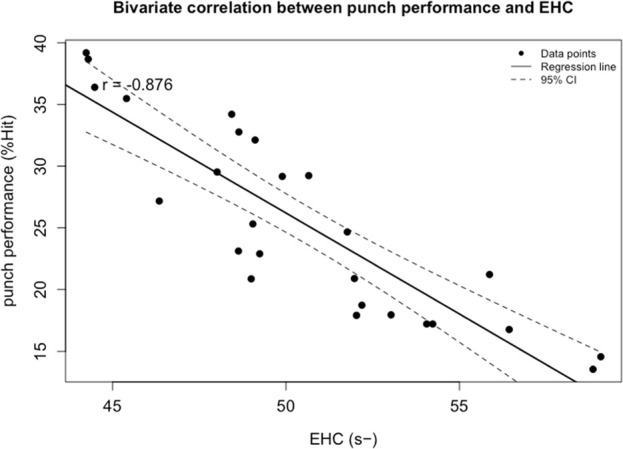
Bivariate correlation between punch performance and variables entered into the regression model: the Eye-Hand Coordination (s). The broken line represents 95% CI. −: lower is better.

**FIGURE 3:**
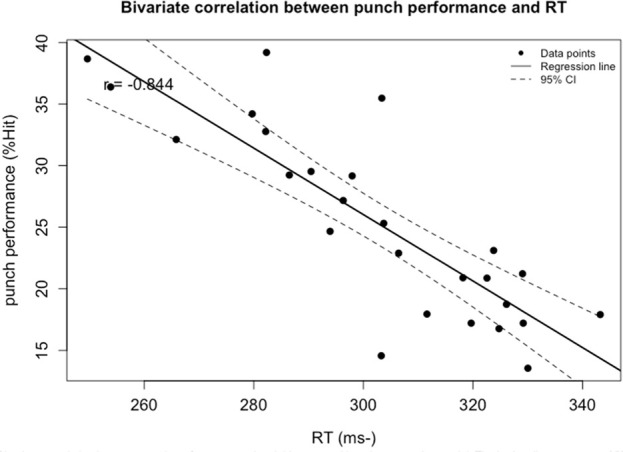
Bivariate correlation between punch performance and variables entered into the regression model: the Reaction Time (ms). The broken line represents 95% CI. −: lower is better.

**FIGURE 4:**
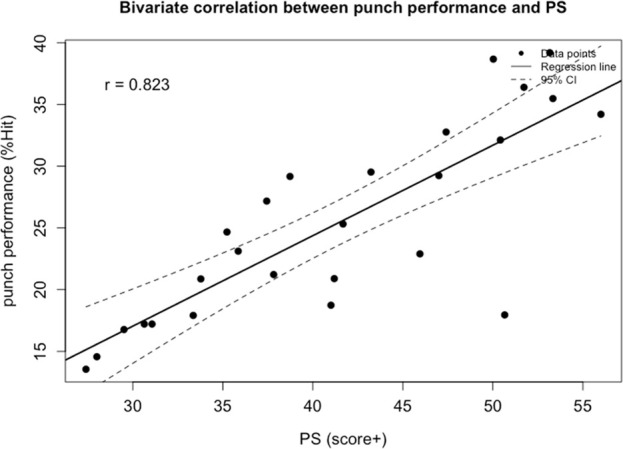
Bivariate correlation between punch performance and variables entered into the regression model: the perception span score+. The broken line represents 95% CI. −: lower is better.

**FIGURE 5:**
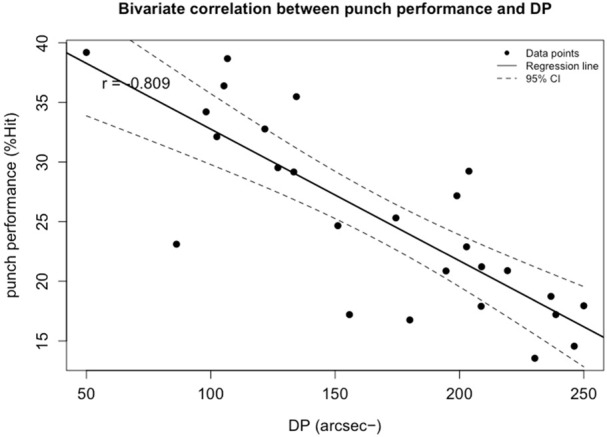
Bivariate correlation between punch performance and variables entered into the regression model: the depth perception score+. The broken line represents 95% CI. −: lower is better.

Multiple stepwise regression analysis confirmed that among all visual abilities, eye-hand coordination, reaction time, peripheral speed, and depth perception significantly predicted boxing performance. The model showed a good fit, with an adjusted *R* ^*2*^ = 0.931 (*p* < 0.001). Table 4 presents the standardized regression coefficients, and Figures 2–5 display the scatter plots of the regression analyses. A *post hoc* power analysis based on effect size (*f* ^*2*^) revealed an effect size of *f* ^*2*^ = 0.33 (G*Power 3.1), which, according to Cohen’s (1988) guidelines, represents a medium-to-large effect. With 26 participants, one tested predictor variable, and an observed effect size of 0.33, the statistical power of the regression model was 0.8. Despite the relatively small sample size, the model demonstrated adequate sensitivity in detecting Type II errors.

## 4 Discussion

This study systematically explores the relationship between visual-motor skills and actual punching performance in female boxing athletes. Among the ten measured visual performance variables, visual clarity (VC), contrast sensitivity (CS), depth perception (DP), perceptual span (PS), multiple object tracking (MOT), reaction time (RT), eye-hand coordination (EHC), and Go/No-Go (GNG) were found to be significantly correlated with strike hit rate (%Hit), with EHC, RT, PS, and DP being the top four predictors. These findings fill a gap in research on visual-motor skills within the field of female boxing, offering new insights into the practical application of enhancing boxing performance through improved visual abilities. Compared to the findings of Wu et al. (2024) on visual predictive abilities in male boxers, this study revealed gender-based differences in the weighting of these variables. Such differences may stem from a range of psychological and physiological factors influenced by sex (Vanston and Strother, 2017; Shaqiri et al., 2018; Mathew et al., 2020).

The present findings are consistent with those of Wu et al. and many previous studies indicating that visual abilities are closely associated with athletic performance in boxing (Wu et al., 2024). The notion that visual abilities play a key role in high-level athletic performance has been supported by numerous studies (Blundell, 1985; Abernethy, 1986; Ciuffreda and Wang, 2004; Burris et al., 2018). The mechanism underlying the relationship between visual abilities and athletic performance can be understood through the information processing model of motor performance (Welford, 1960; Erickson, 2018; Erickson, 2021). According to this model, athletic performance is regulated by three sequentially operating central processing mechanisms: the perceptual mechanism, the decision-making mechanism, and the effector mechanism. The perceptual mechanism integrates and interprets inputs from multimodal receptors (e.g., visual, vestibular, auditory, and tactile), identifies task-relevant stimuli, and suppresses distracting information, thereby constructing a clear representation of the external environment. Processed information is then transmitted to the decision-making mechanism, which rapidly generates or inhibits motor response strategies by integrating prior motor experience with environmental cues. Finally, the decision outcome is executed by the effector mechanism, which precisely controls the timing and manner of actions via activation of the motor cortex and downstream neural circuits. Within this dynamic closed-loop system, the perceptual and decision-making mechanisms interact and update in real-time to adapt to the rapidly changing demands of competition. This model thus provides a theoretical foundation for understanding how visual abilities underpin rapid recognition, accurate judgment, and response execution in boxing (Welford, 1960; Erickson, 2018; Erickson, 2021).

Additionally, this study found distinct differences between male and female boxers in how visual performance metrics predict athletic outcomes. Previous research has shown that male boxers tend to process visual information more rapidly and utilize depth perception more effectively, which gives them an advantage in a sport requiring swift reactions (Ferreira et al., 2017; El Moutaraji et al., 2021; Mathew et al., 2020). In contrast, female boxers may demonstrate superior fine control in visual and motor domains and are more likely to adopt strategic information processing approaches, relying on other cognitive functions to compensate for relatively weaker basic visual abilities (Liutsko et al., 2020; Nicholson and Kimura, 1996). Therefore, the importance of specific visual performance indicators differs between male and female boxers, reflecting possible variations in perceptual and decision-making strategies employed during competition.

The application of sports vision in combat sports such as boxing has been extensively studied, and the relationship between athletes’ performance and visual abilities has been well established (Wu et al., 2024; Chen et al., 2016; Mahlangu et al., 2025; Hijazi, 2013). These findings are consistent with the results of the present study, which indicate that EHC plays a crucial role in boxing. The skill of coordinating eye and body movements is referred to as visuomotor reaction time, defined as the time interval between the appearance of a visual stimulus and the completion of a motor response (Erickson, 2022a). This process forms the foundation for boxers to execute effective actions, respond quickly, and make timely decisions.

In this study, EHC was identified as the most important visual ability variable among female boxers. This aligns with the findings of Chen et al. (2016), who reported that perceptual and motor performance in combat sports athletes vary depending on the specific demands of the discipline. The primary objective for a boxer during a match is to deliver clean punches to the opponent while avoiding counterattacks (Guidetti et al., 2002). This requires visually monitoring the opponent, receiving dynamic visual information, reacting quickly, and executing numerous offensive and defensive maneuvers (Davis et al., 2013; Slimani et al., 2017). From this perspective, the visual-motor processes in boxing are consistent with the definition of eye-hand coordination, which explains why EHC emerged as a key component of visual ability in this study. This also accounts for the positive correlation observed between GNG (Go/No-Go) performance and punching accuracy. Moreover, both EHC and GNG assessments involve rapidly touching or refraining from touching illuminated targets on a screen based on their location, with EHC emphasizing precision accuracy, and GNG focusing on the inhibition of responses to red dots. This method closely aligns with the hand-eye coordination tasks inherent to boxing. A similar perspective was demonstrated in Mahlangu’s (2025) comparative study of visuospatial intelligence between non-athletes and amateur boxers, which employed comparable tasks. Although previous studies have generally emphasized RT as a core visual ability variable for boxers, this study found that EHC had a higher predictive weight than RT in relation to punch hit rate (%Hit) among female boxers. This conclusion is consistent with the semiparametric modeling results reported by Burris et al. (2018), based on data from 2,317 athletes assessed using the Nike SPARQ Sensory Station (Burris et al., 2018). Prior research has also shown that women exhibit a significant advantage in sequencing hand movements (Nicholson and Kimura, 1996). This phenomenon may be closely related to the superiority of the female neuromotor system in fine coordination and movement accuracy (Liutsko et al., 2020), which, in the context of boxing, translates into a competitive edge in the continuity and precision of strike placement, even though single-punch explosive speed may be lower than that of males. However, Swathi et al. (2023), in a study assessing hand-eye coordination in college students through a mirror-tracing task, found higher efficiency indices in males. Yet, this study had two notable limitations: first, the participants were non-athletes; second, the experimental task did not simulate complex athletic scenarios. These factors fundamentally differentiate their conclusions from those of the present study, which is grounded in performance demands specific to competitive boxing.

Reaction time and response time (movement time) are widely regarded as classical indicators of the efficiency and effectiveness with which an individual acquires motor skills (Magill and Anderson, 2010). In the present study, RT remains one of the key visual variables among female boxers. As observed by Stanley et al. (2018), experienced boxers are capable of delivering punches within 402–405 milliseconds, a time frame that necessitates extremely rapid reactions and evasive maneuvers (Stanley et al., 2018). Nevertheless, there appears to be a noticeable decline in the extent to which female boxers rely on RT compared to previous findings. Similar trends have been observed in other combat sports studies that align with the present results (El Moutaraji et al., 2021; Korobeynikov et al., 2025; Ferreira et al., 2017). Male boxers are generally more inclined to adopt fast-reaction strategies, characterized by lower decision thresholds and greater decisiveness when capturing fleeting visual cues. While this strategy enhances reaction speed, it may sometimes compromise accuracy. Research in tracking tasks suggests that men tend to have an advantage in rapid decision-making, converting visual information into motor actions more directly (Mathew et al., 2020). In practical boxing scenarios, this manifests as a tendency among male athletes to initiate quick offensive or defensive actions to gain an advantage. In contrast, female boxers tend to be more cautious in attention allocation and response strategies. They often prioritize situational awareness and the integration of multiple cues, opting for a relatively conservative approach that ensures precision before initiating action. This perspective is supported by Demchenko et al. (2020), who noted that tactical training in female boxing must differ significantly from that of their male counterparts (Demchenko et al., 2020).

In this study, perceptual span emerged as the third most important visual ability variable among boxers. This indicator primarily evaluates an athlete’s ability to recognize and process objects quickly and across a broad visual field using binocular vision. It tests a boxer’s capacity to retain target and positional information within a defined area and to mentally reproduce the corresponding visual patterns essentially, the ability to encode and recall visual spatial configurations. Erickson (2018) categorized this indicator as part of the transformation phase in the visual information processing model (Erickson, 2018). Consistent with the present findings, Burris et al., 2018 reported that perceptual-motor abilities, including perceptual span, significantly predicted on-field performance among professional baseball players (Burris, 2018). In boxing, this ability is reflected in an athlete’s capacity to effectively utilize patterns in an opponent’s behavior to predict future actions, thereby improving decision-making accuracy. This predictive capacity is a major differentiating factor between boxing experts and novices (Bu, 2022; Zhang et al., 2022; Martínez de Quel and Bennett, 2019; Borysiuk et al., 2024). However, compared with prior studies, the importance of perceptual span appears to be amplified in the present research. This phenomenon may be attributed to the rapid development of spatial working memory during early adolescence in females (Gathercole, Pickering, Ambridge and Wearing, 2004), as well as the earlier maturation trajectory commonly observed in female cognitive development.

In the present study, depth perception was also identified as an important visual ability variable among female boxers. Depth perception reflects an athlete’s ability to rapidly and accurately determine the distance of objects in front of them and to understand spatial relationships within their environment (Elmurr, 2011). Accurate depth judgment facilitates navigation, precise timing, and the correct prediction of potential collisions (Erickson et al., 2011; Erickson, 2021). This is particularly crucial in boxing, where athletes must not only accurately assess the opponent’s spatial position and distance but also clearly perceive the state and trajectory of the opponent’s punches in order to respond with precise counterattacks (Davis et al., 2013; Slimani et al., 2017; Davis et al., 2018; Martínez de Quel and Bennett, 2019). These demands strongly support the relevance of depth perception in boxing performance. However, in the present study, the relative importance of the depth perception indicator was found to be lower among female athletes, which may be attributed to inherent gender-based differences in visuospatial abilities. In a study by Gavazzi et al. (2022), functional magnetic resonance imaging (fMRI) was used to examine brain activation patterns in male and female participants during basic visuospatial tasks (Gavazzi et al., 2022). The results showed significantly greater activation in the primary visual cortex among males, suggesting a male advantage in processing spatial cues. These findings provide a plausible neurophysiological explanation for the observed gender difference in the predictive power of depth perception in this study.

Among the other visual ability variables, female boxers’ %Hit was strongly correlated with MOT, moderately correlated with CS and VC, and not correlated with near-far quickness (NFQ) and target capture (TC). This differs from the findings of Burris et al. (2018), who reported that superior performance in TC and NFQ among baseball players was associated with a reduced likelihood of strikeouts (Burris et al., 2018). The discrepancy can be attributed to the nature of the two sports. Baseball players compete in significantly larger spaces and must track both the ball and the dynamic movements of other players, which involve rapid, multidirectional shifts over short and long distances. In contrast, boxers compete in a confined area of approximately 16 by 18 feet and focus primarily on the opponent and referee in close range. Therefore, rapid focal length adjustment, target acquisition, and multi-target tracking may be less critical for boxing performance.

In this study, VC and CS showed only weak correlations with punching performance among female boxers. VC mainly reflects static visual acuity, while CS, as a form of static vision, represents the visual system’s ability to distinguish objects and their background under varying luminance conditions. Both measures reflect general foundational visual capabilities (Elmurr, 2011). Although Laby et al. (2019) reported a significant relationship between visual acuity, contrast sensitivity, and batting performance in professional baseball players, this finding contrasts with the present study (Laby et al., 2019). Nascimento et al. suggested that while good visual abilities are essential for acquiring information in motion, the specific visual skills required may differ depending on the demands of each sport (Nascimento et al., 2020). In high-speed, reaction-intensive sports like boxing, the influence of VC and CS on performance may be less prominent than expected, given the rapid movements and high variability of actions, which could diminish the relative contribution of these basic visual skills.

In contrast, the MOT task in this study assessed boxers’ ability to simultaneously track multiple moving targets within a given timeframe, which also required sustained attention and strategic allocation of attentional resources. During competition, boxers must maintain high levels of concentration while simultaneously monitoring multiple cues such as the opponent’s upper body, chin, glove movements, and position within the ring. Thus, perceptual range, supported by effective attentional distribution strategies, constitutes a key cognitive skill for boxers (Russo and Ottoboni, 2019). Interestingly, Wu et al. (2024) found only a moderate correlation between MOT and punching performance in male boxers (Wu et al., 2024). Gülsoy and Erhan, 2024, however, reported that female athletes outperform their male counterparts in attention-related tasks, which supports the current findings (Gülsoy and Erhan, 2024). This advantage may stem from female boxers’ greater capacity for broad information acquisition, more cautious information filtering, and more balanced multitask attention allocation in multi-target situations (Elshorbagy et al., 2024; Abbas and Jeong, 2024).

In conclusion, this study demonstrates the importance of visual-motor skills in female boxing. Visual-motor abilities are widely recognized as sustainable skills (Appelbaum and Erickson, 2016; Shekar et al., 2021; Erickson, 2022b), meaning that through long-term, systematic training, athletes can significantly enhance their visual-motor coordination, reaction speed, and perceptual range. Moreover, these improved visual-motor skills can be effectively translated into better athletic performance (Lochhead et al., 2024). However, different aspects of visual-motor skills vary in their impact on performance (Presta et al., 2021). Based on the findings of this research, we aim to offer new perspectives and methods for training and strategizing visual skills specifically for female boxers, ultimately helping to elevate their competitive level.

## 5 Limitations

This study has several limitations. First, the small number of participants is a major limitation. Only a few female amateur boxers met the inclusion criteria for the study, which could affect the generalizability and statistical power of the results. As a result, some potential relationships between variables may not have been fully explored. Second, the participants were all female amateur boxers, and the findings of this study are primarily based on this specific group. Therefore, applying the conclusions of this study to other sports may be limited. Additionally, the study did not include a control group, so comparisons with other sports or non-athlete groups could not be made, which limits the broader comparison of the impact of visual abilities on boxing performance. Lastly, although this study used highly reliable testing tools and methods, the variability in boxing matches means that unaccounted external factors, such as psychological state and training level, could influence performance. Future research should expand the sample size and include a control group to validate the causal relationship between visual abilities and boxing performance, as well as explore other potential influencing factors.

## 6 Conclusion

This study investigates the relationship between visual-motor skills and punching performance in female boxers. Among the visual ability variables, visual clarity, contrast sensitivity, depth perception, perceptual span, multiple object tracking, reaction time, eye-hand coordination, and Go/No Go were found to be associated with punching accuracy. The level of these visual abilities affects boxers’ punching performance during competition. In particular, reaction time, eye-hand coordination, perceptual span, and depth perception were identified as key visual abilities for female boxers, and improvements in these areas may enhance their punching performance. Additionally, this study revealed significant gender differences in the distribution of visual ability importance. Male boxers tend to adopt fast-response strategies and exhibit advantages in the speed of visual processing, whereas female boxers demonstrate superior visual precision and fine motor control. Based on these findings, this study recommends incorporating visual ability assessments and training programs specifically tailored for female athletes into boxing training. When designing visual training methods, the unique demands of boxing should be carefully considered to ensure the training effectively enhances female boxers’ in-ring performance. Additionally, the study highlights the impact of visual-motor coordination and other visual skills on punching accuracy and reaction speed, underscoring the critical role of visual training in improving the competitive level of female boxing athletes. Future research should further investigate the relationship between visual abilities and defensive performance in boxing, as well as the effectiveness of visual training on in-competition performance. Moreover, future studies should include a broader range of variables to more comprehensively characterize boxing performance and validate the findings through more refined analytical approaches.

## Data Availability

The original contributions presented in the study are included in the article/supplementary material, further inquiries can be directed to the corresponding author.
