# Supplementary material for: The relationship between visual ability assessment and competitive boxing performance in female amateur boxers
Source: Front Physiol. 2025 Aug 6;16:1639227. doi: 10.3389/fphys.2025.1639227 (PMC12364887; doi:10.3389/fphys.2025.1639227)
Supplement: Supplementary file 1 [file DataSheet1.pdf]

Supplementary Material

TABLE 1 Detailed description of motor visual ability test

Test Indicators	Detailed Methods	Evaluation Criteria
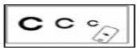 Visual Clarity VC Task	The participant holds a mobile device and stands 3 meters away from a tablet screen. They judge the direction of the gap in a C-shaped figure displayed on the tablet and swipe the corresponding direction on the mobile device. Monocular vision is tested first for the left and right eyes, followed by binocular vision.	The smaller the figure size at which the participant can accurately judge the direction, the better. The test metric is expressed in logMAR units, where lower values indicate better performance. A 5-point scoring system is used, with 5 being the highest score.
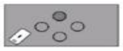 Contrast Sensitivity CS Task	The participant holds a mobile device and stands 3 meters from the tablet. Four black circles appear on the tablet screen; one circle, randomly oriented, contains concentric circles of varying shades. The participant must identify this circle and swipe in the corresponding direction on the mobile device.	As accuracy improves, the contrast within the concentric circles becomes less distinct. The test metric is measured in logCS (log Contrast Sensitivity), with higher values indicating better sensitivity.
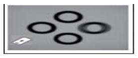 Depth perception DP task	Standing 3 meters from the tablet, the participant sees four black circles on the screen; one randomly displays a stereoscopic effect. The participant must find this circle and swipe in the corresponding direction on the mobile device. Binocular vision is tested first, followed by separate tests for the right and left eyes.	As accuracy increases, the contrast and stereoscopic effect of the target circle become less pronounced. The test metric is in arcseconds (arcsec), where smaller values indicate better stereoscopic vision.

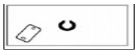 Near-far Fast N/FQ Mission	The participant stands 3 meters from the tablet, holding the mobile device so that its top is 40 cm below the bottom of the tablet screen. During the test, C-shaped figures alternate between the tablet (far) and mobile device (near). The athlete switches focus between near and far every 30 seconds to judge the gap direction, then quickly swipes in the corresponding direction on the mobile device.	Faster judgment speed and higher directional accuracy are preferable. Test metrics include the number of swipes within 30 seconds (higher is better) and reaction times for near and far focus shifts measured in milliseconds (ms), where lower values indicate faster responses.
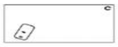 Target acquisition TC mission	The participant holds a mobile device and stands 3 meters from the screen, aligning the blue reference line in the center of the screen with their line of sight, focusing on the center point. C-shaped figures randomly appear in the four corners of the screen; the participant judges the gap direction and swipes the corresponding direction on the mobile device.	Reaction times measured in milliseconds (ms); faster speeds correspond to better performance.
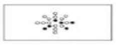 Perception span PS task	The participant stands 60 cm from the tablet screen, with eyes level to the center of the screen. Radial circles emanate from the center, some of which briefly flash black dots at their centers. The athlete must identify and tap the circle containing the black dot.	The number of circles and black dots increases continuously across a wider range. Scores are based on the cumulative number of correct identifications, with higher scores indicating better performance.
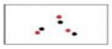 Multi-target tracking MOT mission	The participant stands 60 cm from the tablet screen, eyes level with the screen center. Several groups of spheres appear, each containing two black spheres. One sphere	Test metrics include the number of correctly tracked targets, tracking speed (degrees per second, °/s), percentage-based scores, and composite scores. Higher

	briefly changes to red then quickly returns to black before rotating randomly clockwise or counterclockwise. After rotation stops, the participant must identify the sphere that initially turned red in each group.	values indicate better performance.
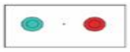 Response Time RT Task	The participant stands 60 cm from the tablet screen, eyes level with the screen center. Radial circles emanate from the center; some flash black dots briefly. The participant must identify and tap the circles containing the black dots.	Similar to above, with increasing numbers and range of circles and black dots, scoring is based on cumulative correct judgments; higher scores indicate better performance.
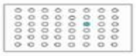 Hand-eye coordination EHC task	The athlete stands 60 cm from a large screen, raising the screen's midline to align with or slightly above the arms to avoid obstructing peripheral vision. The screen displays 8 columns by 10 rows of hollow rings. One ring randomly changes to blue-green, and after the participant clicks it, another ring appears at a random position. The goal is to click as many as possible quickly and accurately within the allotted time.	Metrics include total time, average reaction time, central region reaction time, and peripheral region reaction time all measured in milliseconds (ms), with lower values indicating better performance.
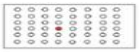 Go/No Go(GNG) task	The athlete stands 60 cm from the large screen, with the screen midline aligned at or slightly above arm level to prevent interference with peripheral target tapping speed. The screen shows 8 columns of circles identical to those in the eye-hand coordination test. Green and red dots appear randomly on the circles; green dots require a	Metrics include overall score, number of correct taps (higher is better), and number of incorrect taps (lower is better).

quick tap, while red dots must not be tapped.

TABLE 2 Description of boxing performance analysis variables.

Variable	Unit	Description
Punches Thrown	Number	Total number of punches thrown
Hit	Number	A punch that hits the target area
Miss	Number	A punch that misses the target area
%Hit	%	Number of hits to the target area as a percentage of total punches thrown

TABLE 3 Descriptive analysis of anthropometrics, visual ability tests, and punch accuracy.

	M±SD N=26
Personal data	
Age(y)	24.69±5.48
Height (cm)	170.81±6.66
Weight (kg)	67.07±7.39
Experience(y)	7.19±2.51
Visual ability variables	
EHC（s-）	50.57±4.15
GNG(scoer+)	6.32±1.40
RT（ms-）	302.82±24.28

PS（score+）	41.21±8.71
DP（arcsec-）	167.90±56.85
MOT（score+）	0.70±0.10
VC（logMAR-）	-0.03±0.08
CS（logCS+）	1.67±0.26
TC（ms-）	271.32±83.19
NFQ（score+）	16.15±3.75
Punch variables	
Punch accuracy (%Hit)	25.25±7.76

VC, visual clarity; CS, contrast sensitivity; DP, depth perception; NFQ, near far quickness; TC, target capture; PS, perception span; MOT, multiple object tracking; EHC, Eye-Hand Coordination; GNG, Go/No Go; RT, reaction time; + = higher is better; − = lower is better.

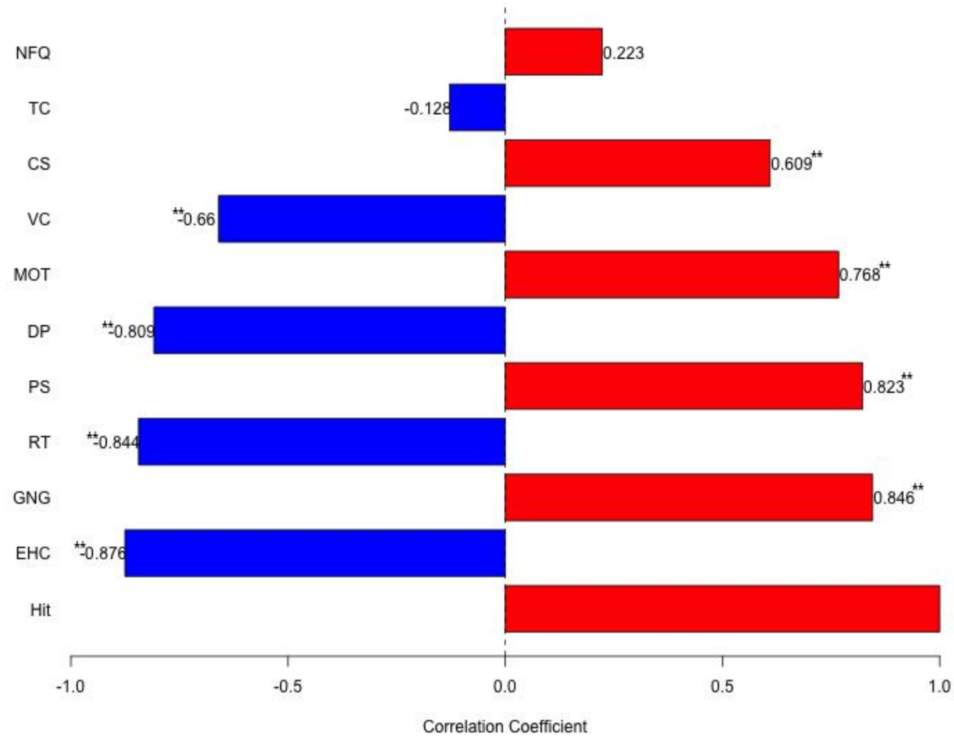

FIGURE 1 Correlation coefficients between boxers' visual ability and punching performance.a b

***p < 0.05.**

****p < 0.01.**

TABLE 4 Standardized regression to explain the punch performance (%Hit).a

	ß	CI for ß	P
EHC	-0.304	-0.931 to -0.206	0.004**
RT	-0.309	-0.152 to -0.045	0.001**
PS	0.231	0.046 to 0.366	0.014*
DP	-0.278	-0.06 to -0.016	0.002**
Modelt fit	Adjusted R2=0.931		

a %Hit = hit rate of punches thrown;EHC, Eye-Hand Coordination ; RT, reaction time;PS,perception span; DP, depth perception; βa = estimated standardized regression coefficient.

*p < 0.05.

**p < 0.01.

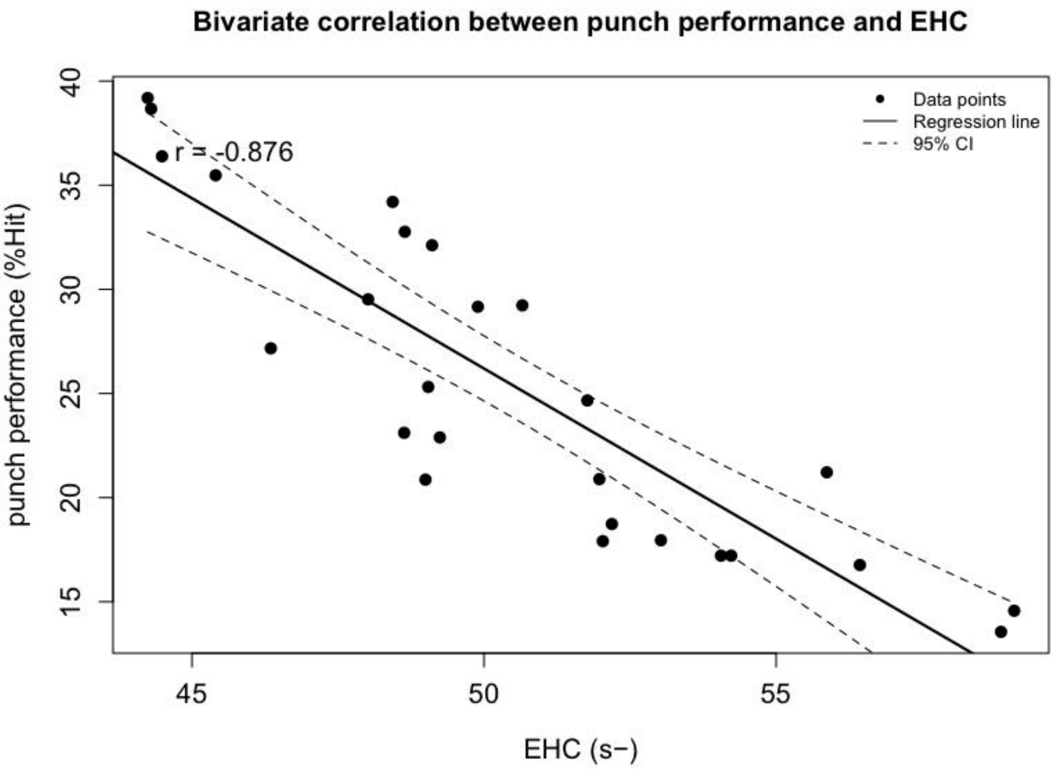

FIGURE 2 Bivariate correlation between punch performance and variables entered into the regression model: the Eye-Hand Coordination (s). The broken line represents 95% CI. −: lower is better.

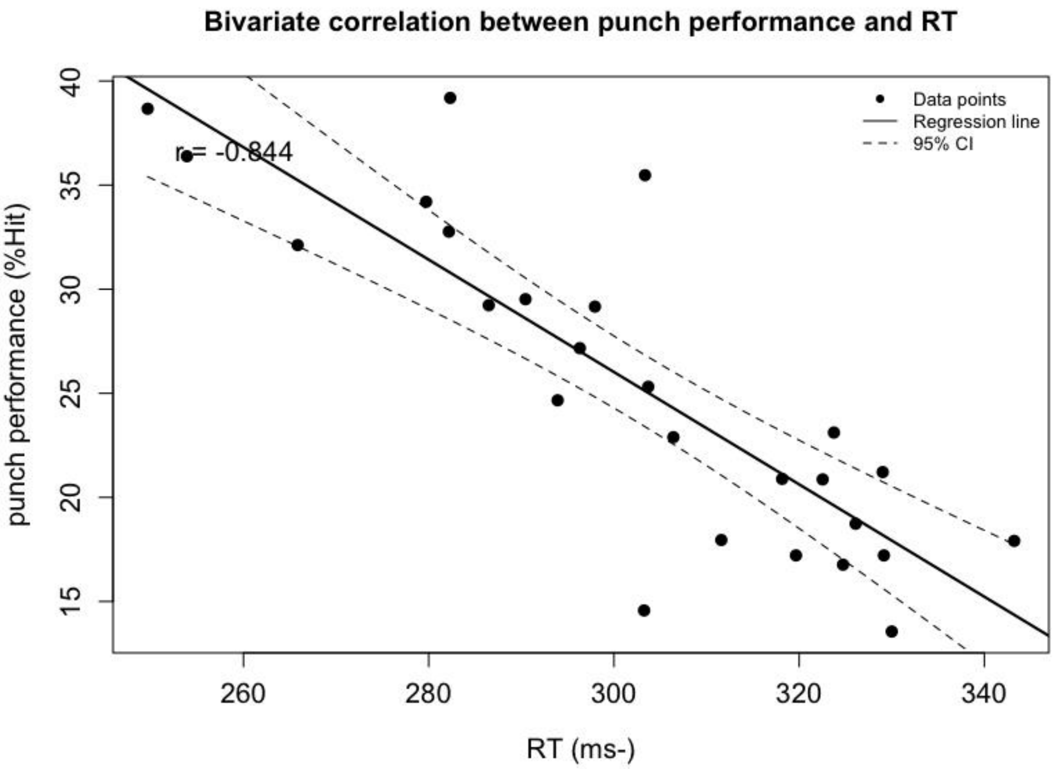

FIGURE 3 Bivariate correlation between punch performance and variables entered into the regression model: the Reaction Time (ms). The broken line represents 95% CI. −: lower is better.

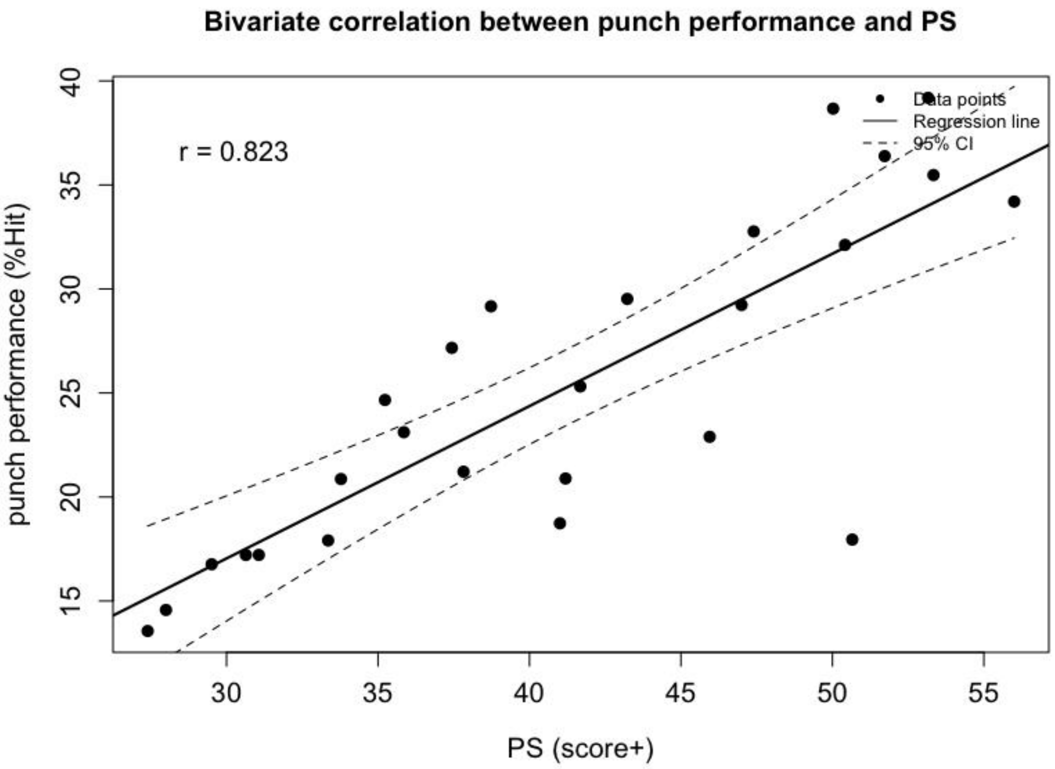

FIGURE 4 Bivariate correlation between punch performance and variables entered into the regression model: the perception span score+. The broken line represents 95% CI. −: lower is better.

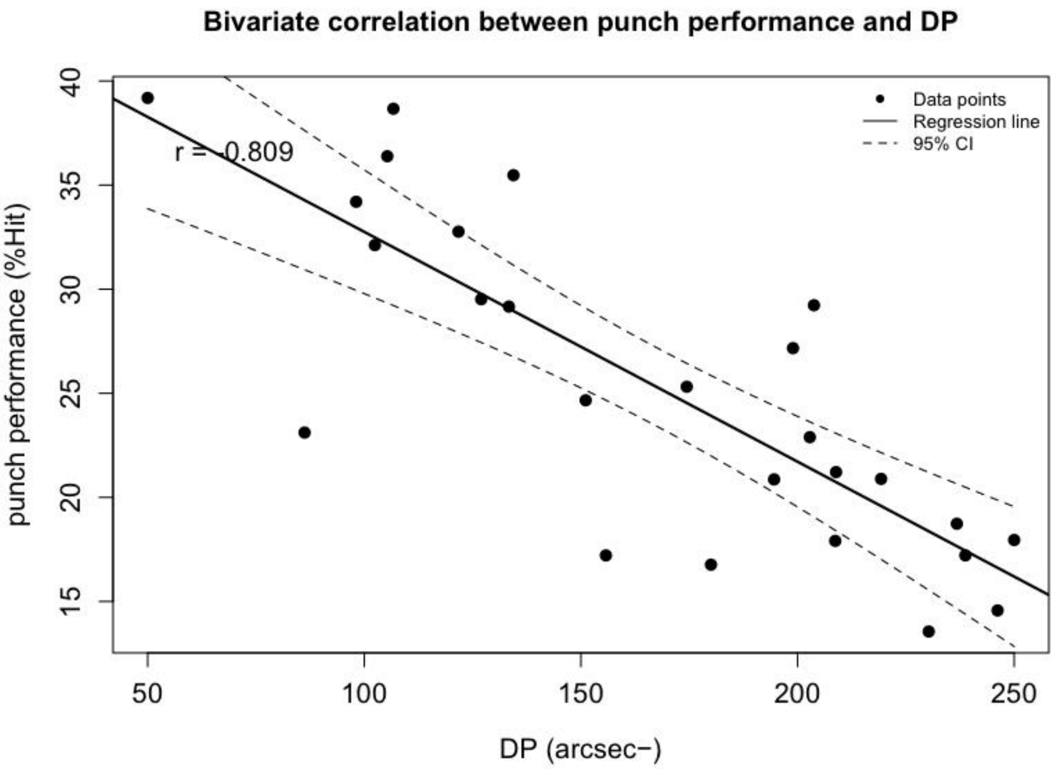

FIGURE 5 Bivariate correlation between punch performance and variables entered into the regression model: the depth perception score+. The broken line represents 95% CI. −: lower is better.
